# Alpha-1-Antitrypsin Enhances Primary Human Macrophage Immunity Against Non-tuberculous Mycobacteria

**DOI:** 10.3389/fimmu.2019.01417

**Published:** 2019-06-26

**Authors:** Xiyuan Bai, An Bai, Jennifer R. Honda, Charles Eichstaedt, Ariel Musheyev, Zhihong Feng, Gwen Huitt, Ronald Harbeck, Beata Kosmider, Robert A. Sandhaus, Edward D. Chan

**Affiliations:** ^1^Division of Pulmonary, Critical Care, and Sleep Medicine, National Jewish Health, Denver, CO, United States; ^2^Academic Affairs, National Jewish Health, Denver, CO, United States; ^3^Division of Pulmonary Sciences and Critical Care Medicine, University of Colorado School of Medicine, Aurora, CO, United States; ^4^Center for Genes, Environment, and Health, National Jewish Health, Denver, CO, United States; ^5^Department of Respiratory Medicine, Xuanwu Hospital, Capital Medical University, Beijing, China; ^6^Department of Thoracic Medicine and Surgery, Temple University, Philadelphia, PA, United States; ^7^Center for Inflammation, Translational and Clinical Lung Research, Temple University, Philadelphia, PA, United States; ^8^Department of Physiology, Temple University, Philadelphia, PA, United States; ^9^Department of Medicine, Rocky Mountain Regional Veterans Affairs Medical Center, Denver, CO, United States

**Keywords:** autophagy, mycobacteria, nuclear factor-kappa B, phagosome-lysosome fusion, serine protease inhibitor

## Abstract

**Rationale:** The association between non-tuberculous mycobacterial lung disease and alpha-1-antitrypsin (AAT) deficiency is likely due, in part, to underlying emphysema or bronchiectasis. But there is increasing evidence that AAT itself enhances host immunity against microbial pathogens and thus deficiency could compromise host protection.

**Objectives:** The goal of this project is to determine if AAT could augment macrophage activity against non-tuberculous mycobacteria.

**Methods:** We compared the ability of monocyte-derived macrophages cultured in autologous plasma that were obtained immediately before and soon after AAT infusion—given to individuals with AAT deficiency—to control an *ex vivo Mycobacterium intracellulare* infection.

**Measurements and Main Results:** We found that compared to pre-AAT infused monocyte-derived macrophages plus plasma, macrophages, and contemporaneous plasma obtained after a session of AAT infusion were significantly better able to control *M. intracellulare* infection; the reduced bacterial burden was linked with greater phagosome-lysosome fusion and increased autophagosome formation/maturation, the latter due to AAT inhibition of both *M. intracellulare*–induced nuclear factor-kappa B activation and A20 expression. While there was a modest increase in apoptosis in the *M. intracellulare*-infected post-AAT infused macrophages and plasma, inhibiting caspase-3 in THP-1 cells, monocyte-derived macrophages, and alveolar macrophages unexpectedly reduced the *M. intracellulare* burden, indicating that apoptosis impairs macrophage control of *M. intracellulare* and that the host protective effects of AAT occurred despite inducing apoptosis.

**Conclusion:** AAT augments macrophage control of *M. intracellulare* infection through enhancing phagosome-lysosome fusion and autophagy.

## Introduction

Non-tuberculous mycobacteria (NTM) are ubiquitous organisms found in water, biofilms present at water-solid interfaces, and soil (1). Certain medical and host phenotypic conditions predispose to NTM lung disease (NTM-LD) but the most common are emphysema and preexisting bronchiectasis (2–8). We previously reported that the frequency of anomalous alpha-1-antitrypsin (AAT) phenotypes (mostly heterozygous) in 100 consecutive subjects with NTM-LD due to rapidly-growing mycobacteria—mostly *Mycobacterium abscessus*—was 1.6-fold greater than the estimated frequency of AAT anomalies in the general U.S. population (9). Addition of exogenous AAT to primary human monocyte-derived macrophages (MDM) that were subsequently infected with *M. abscessus* reduced the bacterial burden although the mechanism remains unknown (9).

It is becoming increasingly clear that AAT deficiency is not only associated with precocious emphysema but also bronchiectasis (10). The greater prevalence of bronchiectasis may be related to the immune-enhancing effect of AAT against bacterial and viral pathogens (9, 11, 12). Bronchiectasis may either be a risk factor for NTM-LD or be the sequela of it; i.e., we reasoned that if AAT deficiency predisposes to repeated airway infections, bronchiectasis may be the denouement (13). Among the nearly 200 species of NTM that have been identified, those that belong to the *Mycobacterium avium* complex (MAC) group are the most common causes of NTM-LD and of the MAC species, *M. intracellulare, M. avium hominissuis*, and *M. chimaera* are the most common offenders. Thus, we undertook a study to determine whether MDM and plasma obtained from AAT-deficient subjects before and after AAT infusion have differential ability to control an *ex vivo M. intracellulare* infection. The most common AAT mutation that results in frank AAT deficiency is the Z mutation due to a point mutation (Glu342Lys) of the normal M-AAT.

## Experimental Methods

### Materials

*Mycobacterium intracellulare* 9141 was obtained from the Clinical Mycobacterial Laboratory at National Jewish Health (NJH). The human monocytic cell line THP-1 was obtained from the American Type Culture Collection (Rockville, MD). Fetal bovine serum (FBS) was purchased from Atlanta Biologicals (Lawrenceville, GA) and heat inactivated at 56°C. Reagents for Middlebrook 7H10 solid agar medium or Middlebrook 7H9 liquid medium were purchased from Difco (Detroit, MI). Interferon-gamma (IFNγ), caspase-3 inhibitor benzyloxycarbonyl-Asp-Glu-Val-Asp-fluoromethylketone (z-DEVD-fmk), BD Vacutainer CPT^TM^ tubes, and the Human Active Caspase-3 Quantikine ELISA Kit were purchased from R&D Systems (Minneapolis, MN). LysoTracker Red DND-99, Cy3-goat anti-rabbit IgG (H+L), and anti-A20 polyclonal antibody were purchased from Thermo Fisher Scientific/Life Technologies (Carlsbad, CA). Monocyte colony stimulating factor (M-CSF), phorbol myristate acetate (PMA), dimethyl sulfoxide (DMSO), 3-methyladenine (3-MA), and pure AAT were purchased from Sigma Chemical Company (St. Louis, MO). Polyclonal rabbit anti-human LC3B and p62 antibodies were purchased from Cell Signaling Technology (Danvers, MA). The TransAM® NFκB p65 kit was purchased from Active Motif (Carlsbad, CA). AAT (Glassia®) was acquired from Kamada Ltd., Israel.

### Human Subjects

Following approval by the Colorado Multiple Institutional Review Board (Protocol Number 14-0755) and informed consent, 10 emphysematous subjects with AAT deficiency were recruited. All have homozygous mutation of the *SERPIN1* gene resulting in the protease inhibitor Z-phenotype (PiZZ) and were undergoing intravenous AAT augmentation therapy, with eight subjects receiving Prolastin® and two Zemaira® at a dose of 60 mg/kg weekly. From each individual, 16 mL of blood was obtained immediately before AAT infusion and another 16 mL within 30 min after infusion completion.

### Measurement of AAT

Plasma AAT concentration was measured by turbidimetry using goat anti-human AAT antiserum with a chemistry analyzer according to manufacturer's instructions (Beckman Coulter AU480).

### Differentiation of Monocyte-Derived Macrophages

Peripheral blood mononuclear cells (PBMC) were isolated from blood collected in CPT^TM^ tubes. The separated plasma were cryopreserved and 4–6 × 10^5^ PBMC/well of a 24-well polystyrene plate were differentiated to MDM with 20 ng/mL M-CSF in RPMI 1640 medium containing 10% FBS and penicillin/streptomycin at 37°C, 5% CO_2_. On day 4 of differentiation, additional fresh 0.25 mL of medium without antibiotics or M-CSF was added to each well. On day 6, the cells were washed with a solution containing equal volumes of RPMI and 1X PBS and incubated overnight before use with RPMI medium without antibiotics supplemented with 1% glutamine and either 10 or 50% (v/v) of human plasma that were obtained before and after AAT infusion. The rationale for including the 50% plasma is that with 10% plasma—the typical amount used in cell culture medium—the AAT levels in the cell culture are well-below the range of normal human plasma.

### Isolation of Primary Human Alveolar Macrophages

We obtained deidentified human lungs not suitable for transplantation from the National Disease Research Interchange (Philadelphia, PA) and the International Institute for the Advancement of Medicine (Edison, NJ). We selected lung donors with a clinical history and X-ray that did not indicate infection and with a PaO_2_/FiO_2_ ratio of >250 mmHg. Alveolar macrophages were isolated as we previously described (14). Briefly, the lung was lavaged with HEPES and 2 mM EDTA and the lavage fluid was centrifuged. The cell pellet was resuspended and plated in DMEM supplemented with 10% FBS, 2 mM glutamine, 100 μg/mL streptomycin, 100 U/mL penicillin, 2.5 μg/mL amphotericin B and 10 μg/mL gentamicin. After 24 h, alveolar macrophages were cultured for 2 days in DMEM with 5% FBS.

### Infection and Quantitation of Cell-Associated *M. intracellulare*

Cultured MDM were infected with *M. intracellulare* at a multiplicity-of-infection (MOI) of 10:1 (mycobacteria:macrophage). After 1 h of infection, the cells were washed to remove unphagocytosed *M. intracellulare*. For the 1 h time point, the cells were lysed, the lysate serially diluted, and plated on 7H10 solid medium to quantify cell-associated *M. intracellulare*. For the 2 and 4 day time points, fresh medium was added after the initial 1 h of infection and wash, and incubated for an additional 2 and 4 days before the cells were washed, lysed, serially diluted, and cultured on 7H10 solid medium (15).

### Co-localization of *M. intracellulare* and Lysosomes

Phagosome-lysosome fusion was analyzed as previously described (16). Construction of green fluorescent protein (GFP)-labeled *M. intracellulare* used the pBCM plasmid as reported (17). Briefly, PBMC obtained before and after AAT infusion were seeded in Nunc Lab-Tek II chamber slides and differentiated into MDM; after washing the cells with RPMI:1X PBS solution, RPMI supplemented with 1% glutamine and either 10 or 50% (v/v) of their autologous plasma was added for 1 h, and then infected with *M. intracellulare*–GFP at a MOI of 10:1 for 6 h. Control cells consisted of MDM from healthy individuals that were left unstimulated or stimulated with 10 U/mL IFNγ. Two hours before the stimulation or infection time was complete, LysoTracker Red DND-99 (50 nM) was added to the cells to identify the lysosomes. After a total of 6 h of stimulation or infection, the cells were fixed, stained, and mounted with ProLong Gold Antifade reagent with DAPI. All cellular images were viewed as a single plane using an inverted epifluorescence microscope with a 40X oil objective (Zeiss PLAN-NEOFLUAR) and a 10X eyepiece to give a total magnification of 400X as well as a lens numerical aperture of 1.3 (Carl Zeiss Axiovert 200 M). Controls for the GFP and Lysotracker Red were performed at the same settings as the experimental samples and no signal from one channel was detected in the other. Fifteen, non-overlapping fields were photographed per condition for each experiment. The percentage of macrophages with evidence of phagosome-lysosome fusion was quantified by counting and then dividing the number of cells that contain co-localization of intracellular *M. intracellulare*–GFP with lysosomes by the number of *M. intracellulare*–GFP infected cells (16).

### Autophagosome Number and Maturation

PBMC obtained before and after AAT infusion were differentiated into MDM in chamber slides, cultured in RPMI medium supplemented with 1% glutamine and either 10 or 50% (v/v) of their respective contemporaneous plasma 1 h prior to infection, infected with *M. intracellulare*–GFP at a MOI of 10:1 for 18 h. The cells were fixed with 4% paraformaldehyde, permeabilized, blocked with blocking buffer (1X PBS, 0.1% Tween 20, 5% (w/v) BSA), and sequentially incubated with anti-LC3B antibody, Cy3-labeled anti-rabbit antibody, and DAPI. LC3B-positive particles were quantified by counting the number of red colored particles per cell as previously described (17, 18). Quantitation of p62 positive particles was performed similarly but in a temporal fashion—i.e., for 3, 6, and 18 h after infection—followed by staining with anti-p62 antibody, Cy3-labeled anti-rabbit antibody, and DAPI.

### Apoptosis

MDM cultured in the presence of 10 or 50% plasma from PiZZ subjects obtained before and after AAT infusion were left uninfected or infected with *M. intracellulare* in chamber slides. After 48 h, the cells were stained by TUNEL (Terminal deoxynucleotidyl transferase dUTP nick end labeling) using the *in situ* cell death detection kit (Roche) and percent apoptotic cells was quantified as previously described (17).

### Cytokines

Supernatants of MDM cultured in the presence of 10 or 50% plasma and infected with *M. intracellulare* for 1 h, 2 and 4 days were quantified for tumor necrosis factor-alpha (TNFα), interleukin-1-beta (lL-1β), lL-8, lL-10, lL-12, IL-6, and IFNγ using HCYTOMAG-60K/MILLIPLEX®MAP Human Cytokine/Chemokine Magnetic Bead Panel-Immunology Multiplex Assay (EMDMILLIPORE Inc., Temecula, CA) with the Luminex MAGPIX instrument (Luminex Inc.).

### Caspase-3 Activity

Activated caspase-3 was quantified by the Human Active Caspase-3 Quantikine ELISA (R&D Systems), following manufacturer's instructions and as reported in a previous study (15).

### p65 Nuclear Factor-Kappa B (NFκB) Binding Assay

Activtion and binding of the p65 subunit of NFκB to its *ci*s-reglatory element was quantified with the TransAM^TM^ NFκB p65 kit (Active Motif, Carlsbad, CA), following manufacturer's instruction and as previously reported (18).

### Statistical Analysis

Replicate experiments are independent, and data are presented as means ± SEM or representative experiment. Group means were compared by repeated-measures ANOVA using Fisher's least significant test or by two-way ANOVA with Bonferroni's *post-hoc* test.

## Results

### Mean Plasma AAT Levels of 10 PiZZ Subjects Before and After a Session of AAT Infusion

**Figure 1:**
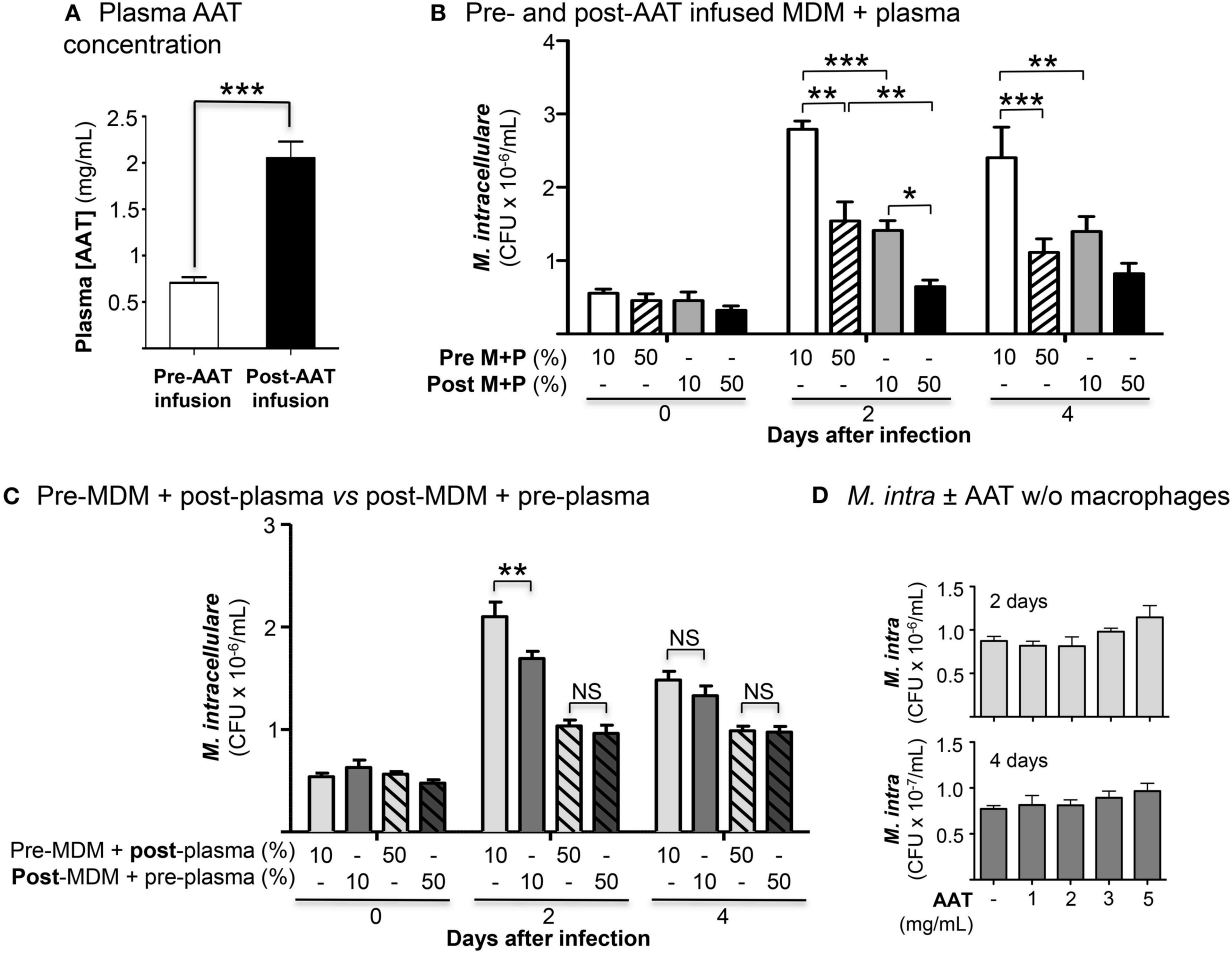
Effects of AAT infusion on macrophage control of *M. intracellulare* infection. **(A)** Mean plasma AAT levels ± SD, measured by nephelometry, pre- and post-AAT infusion of 10 PiZZ subjects. The normal range for plasma AAT at National Jewish Health is 0.72–1.92 mg/mL. **(B)** Quantitation of *M. intracellulare* in MDM cultured in plasma, both obtained before and after AAT infusion from PiZZ patients. Data shown are the mean ± SEM of cells from 10 subjects. **(C)** Pre-AAT infused MDM + post-AAT infused plasma (10 or 50%) vs. post-AAT infused MDM + pre-AAT infused plasma (10 or 50%) were infected with *M. intracellulare*. One hour, 2 and 4 days after infection, the cells were washed, lysed, and *M. intracellulare* quantified. Data shown are the mean ± SEM of cells from five subjects. **(D)** AAT (1, 2, 3, or 5 mg/mL) was incubated with a liquid culture of 5 × 10^5^ *M. intracellulare*/mL without macrophages for 2 and 4 days and *M. intracellulare* quantified. Data shown are the mean ± SD of three independent experiments. ^*^p < 0.05, ^**^p < 0.01, ^***^p < 0.001, NS, non-significant; Pre M+P, pre-AAT infused MDM + plasma; Post M+P, post-AAT infused MDM + plasma; AAT, alpha-1-antitrypsin; *M. intra, M. intracellulare*.

We enrolled 10 PiZZ subjects with emphysema who were receiving weekly AAT augmentation therapy. The mean plasma AAT level in the 10 subjects was 0.77 mg/mL immediately before a session of AAT infusion and was 2.05 mg/mL within 30 min after completion of AAT infusion (Figure 1A) (normal reference range of 0.72–1.92 mg/mL).

### AAT Infusion Enhances Macrophage Control of *M. intracellulare* Infection

The number of cell-associated *M. intracellulare* were quantified 1 h, 2 and 4 days after infection of pre- and post-AAT infused MDM cultured in 10 or 50% contemporaneous plasma. At 1 h after infection, there was no significant difference in the number of viable *M. intracellulare* isolated among all the conditions (Figure 1B). With MDM and plasma obtained before AAT infusion, there was a significant reduction in the number of viable *M. intracellulare* in MDM incubated with 50% plasma compared to cells cultured in 10% plasma (Figure 1B, bar 1 vs. 2 for Days 2 and 4). The number of *M. intracellulare* was further reduced in MDM and plasma obtained after AAT infusion, with the most reduction seen with 50% plasma (Figure 1B, bars 1 and 2 vs. 3 and 4, respectively, for Days 2 and 4). In determining whether post-AAT infused plasma or MDM plays a greater role in limiting the bacterial burden, we compared pre-AAT infused MDM + post-AAT infused plasma vs. post-AAT infused MDM + pre-AAT infused plasma. Post-AAT infused MDM had a modestly greater host-protective effect than post-AAT infused plasma but only at day 2 (Figure 1C). These findings indicate that both post-AAT infused MDM + plasma are required to have optimal effect in limiting the intracellular bacterial burden (compare Figures 1B,C). To assess whether AAT itself, without macrophages, may inhibit growth of the NTM, liquid cultures of 5 × 10^5^/mL of *M. intracellulare* were incubated with 7H9 medium alone or with 1, 2, 3, or 5 mg/mL of AAT for 2 and 4 days and *M. intracellulare* quantified. We found that AAT had negligible *increase* in *M. intracellulare* growth in the absence of macrophages (Figure 1D).

### AAT Infusion Enhances Macrophage Phagosome-Lysosome Fusion

**Figure 2:**
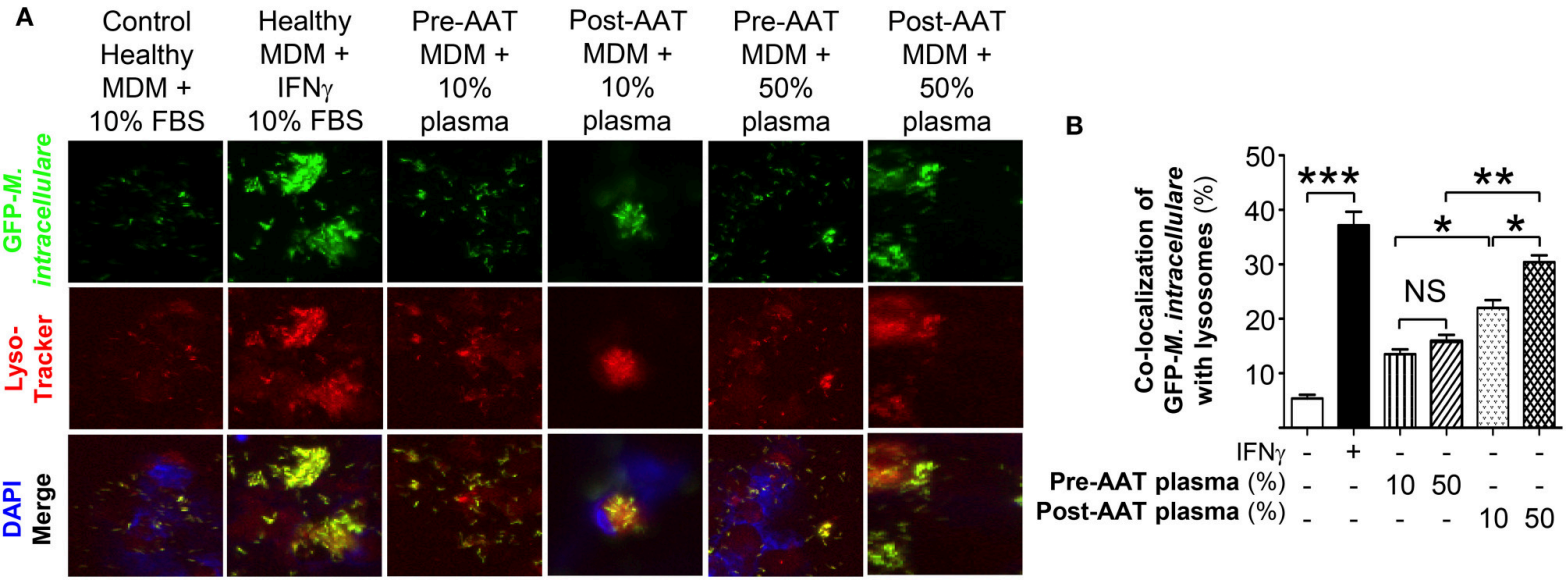
Quantitation of phagosome-lysosome fusion in MDM infected with *M. intracellulare*. **(A)** Monocytes obtained from PiZZ subjects pre- and post-AAT infusion were differentiated into macrophages and incubated with contemporaneous 10% and 50% plasma, and infected with GFP-*M. intracellulare* for 6 h. The cells were then stained for lysosomes with LysoTracker Red®, fixed, and stained with ProLong Gold Antifade Reagent with DAPI and placed in the dark at 4°C until analysis by fluorescent microscopy. Controls included MDM from healthy individuals incubated with 10% FBS, infected with *M. intracellulare*–GFP ± 10 U/mL IFNγ for 6 h. **(B)** The percentage of MDM with co-localization of *M. intracellulare*–GFP and lysosomes was calculated based on the number of cells with co-localization divided by the number of GFP-*M. intracellulare* infected cells. Data represent the mean ± SEM of cells from five subjects performed in duplicates. ^*^p < 0.05, ^**^p < 0.01, ^***^p < 0.001, NS, non-significant.

Co-localization of phagocytosed *M. intracellulare*-GFP with lysosomes was quantified in infected macrophages stained with LysoTracker Red® (16). For baseline and positive controls, PBMC from five healthy individuals were differentiated into MDM in the presence of heat (56°C)-inactivated 10% FBS, a temperature known to inactivate AAT (19), and infected with *M. intracellulare* alone or with 10 U/mL IFNγ, respectively. Compared to MDM infected with *M. intracellulare* alone, there was significantly more phagosome-lysosome fusion in *M. intracellulare*-infected MDM stimulated with IFNγ (Figure 2). There was also increased phagosome-lysosome fusion with MDM and plasma obtained post-AAT infusion (with either 10 or 50% plasma) compared with MDM plus their respective plasma concentrations obtained pre-AAT infusion (Figure 2).

### AAT Infusion Increases Autophagosome Formation and Maturation

**Figure 3:**
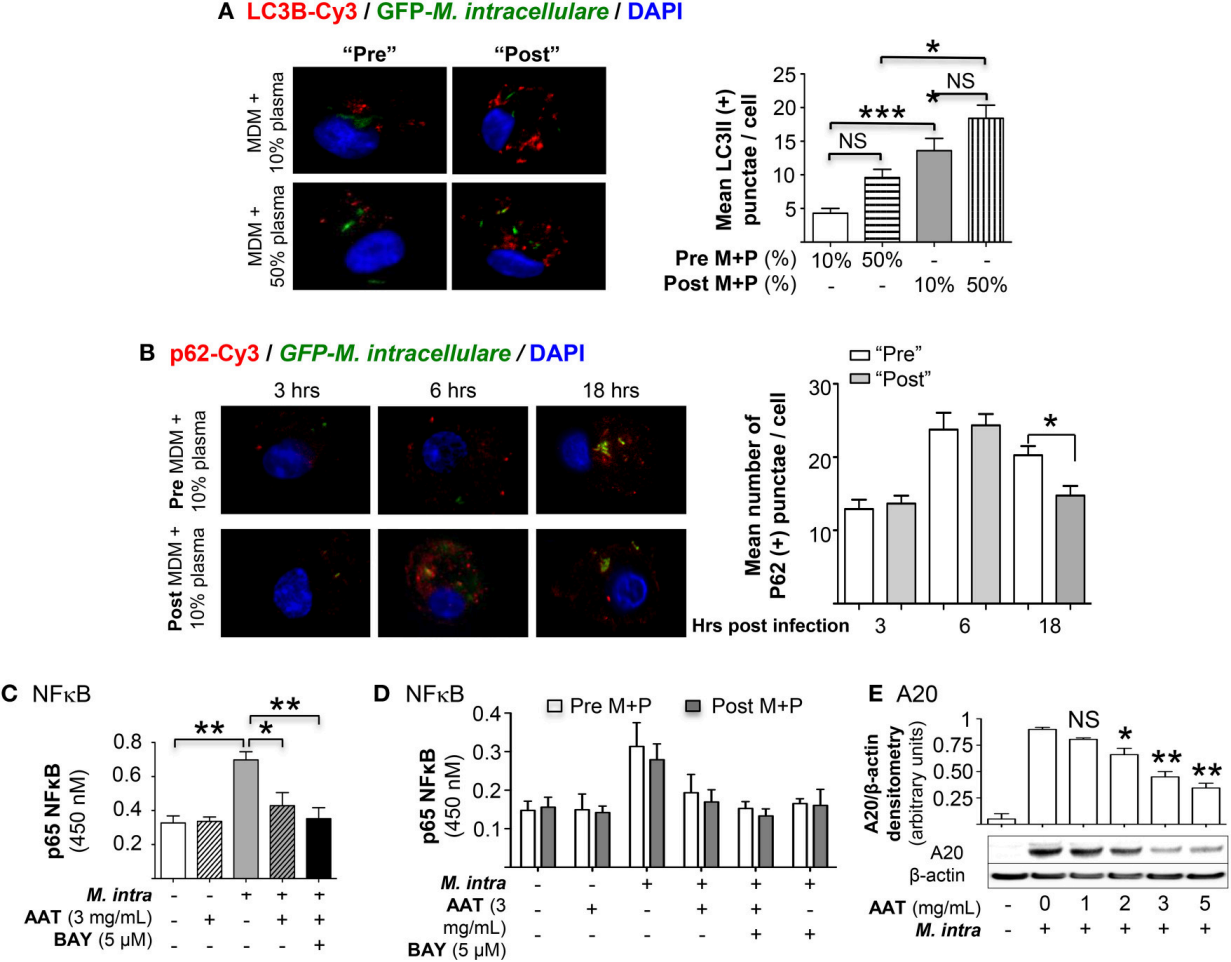
Quantitation of autophagy in MDM infected with *M. intracellulare*. **(A)** MDM plus 10 or 50% plasma obtained pre- and post-AAT infusion were infected with *M. intracellulare*–GFP for 18 h, fixed, and immunostained for LC3B. The number of LC3B-positive intracellular particles was quantified by averaging the number of such particles per cell with 200 cells counted per condition for each experiment. Data represent the mean ± SEM of cells from six subjects performed in duplicates. **(B)** MDM plus 10 or 50% plasma obtained pre- and post-AAT infusion were infected with GFP-labeled *M. intracellulare* for 1 h, washed, and incubated for an additional three, 6 and 18 h. The cells were then fixed and immunostained for p62 and nuclei. The number of p62-positive intracellular particles was quantified by counting the number of p62-Cy3 positive particles per cell with 200 cells counted per condition for each experiment. Data represent the mean ± SEM of cells from three subjects performed in duplicates. **(C)** Quantitation of p65 subunit of NFκB binding to its consensus oligonucleotide in THP-1 cells with *M. intracellulare* infection alone or with 3 mg/mL AAT or 5 μM of the IκBα kinase inhibitor BAY 11-7082 (BAY) for 6 h. Data represent the mean ± SEM of three independent experiments performed in duplicates. **(D)** Quantitation of p65-NFκB binding in pre- and post-AAT infused MDM incubated in 10% contemporaneous plasma and infected with *M. intracellulare* alone or with AAT (3 mg/mL), BAY 11-7082 (5 μM), or both for 6 h. Data represent the mean ± SEM of cells from three subjects performed in duplicates. **(E)** Western blot of A20 protein expression in THP-1 cells with *M. intracellulare* infection alone or with AAT for 24 h. Immunoblot shown is representative of three independent experiments. A20 expression was semi-quantified, reported as the mean densitometry from three independent experiments. Pre M+P, pre-AAT infused MDM + plasma; Post M+P, post-AAT infused MDM + plasma; *M. intra, M. intracellulare*. ^*^p < 0.05, ^**^p < 0.01, ^***^p < 0.001, NS, non-significant.

Autophagosome formation was quantified by immunofluorescent staining with anti-LC3B antibody of MDM incubated in 10 or 50% plasma obtained pre- or post-AAT infusion and infected with *M. intracellulare*–GFP. For MDM in 50% plasma, whether obtained pre- or post-AAT infusion, there was a trend toward increased autophagosome number as compared to their respective MDM in 10% plasma (Figure 3A, bars 1 vs. 2 and 3 vs. 4, respectively). However, MDM in 10 or 50% plasma obtained post-AAT infusion had significantly greater number of autophagosomes compared to their respective MDM and plasma obtained pre-AAT infusion (Figure 3A, bars 1 vs. 3 and bars 2 vs. 4).

To assess autophagosome maturation, we temporally quantified by immunofluorescence the number of particles positive for the multifunctional autophagosomal protein p62 in MDM infected with *M. intracellulare*. While there was an increase in the number of p62-positive autophagosomes from 3 to 6 h after infection with MDM plus plasma obtained either pre- or post-AAT infusion, there was no difference between the pre- vs. post-AAT infused conditions (Figure 3B). However, at 18 h after infection, there was a modest but significant reduction in the number of p62-positive autophagosomes in the MDM and plasma obtained post-AAT infusion compared to pre-AAT infused MDM + plasma (Figure 3B), indicating increased autophagosome maturation with *M. intracellulare*-infected post-AAT-infused MDM and plasma.

A possible mechanism by which AAT enhances autophagy is *via* inhibition of NFκB as previously reported for *Mycobacterium tuberculosis* (17). Indeed, M-AAT is known to inhibit NFκB activation (12, 20, 21), accounting for its anti-inflammatory effects (12, 22, 23). We confirmed this using pure AAT since AAT products administered to patients also contain other plasma components. THP-1 cells were infected with *M. intracellulare* ± AAT (3 mg/mL) for 6 h, followed by p65-NFκB binding assay using a consensus oligonucleotide sequence for NFκB and an antibody specific for the p65 subunit of NFκB. As shown Figure 3C, *M. intracellulare* induced p65-NFκB binding to its oligonucleotide and AAT inhibits this binding. To determine the effects of AAT on *M. intracellulare*-induced NFκB activation in pre- and post-AAT infused MDM incubated with 10% contemporaneous plasma, the relevant cells were infected with *M. intracellulare* alone or with 3 mg/mL AAT, 5 μM BAY 11-7082 (IκBα kinase inhibitor), or both for 6 h. Similar to that seen with THP-1 cells, *M. intracellulare* induced p65-NFκB binding whereas either AAT or BAY 11-7082 significantly inhibited this binding (Figure 3D). However, unexpectedly, there was no difference in p65-NFκB binding between the pre- and post-AAT infused MDM with *M. intracellulare* infection—albeit there was an insignificant trend toward decreased p65-NFκB binding in post-AAT infused MDM (Figure 3D). Since NFκB is a transcriptional activator of A20, a deubiquitinating enzyme known to inhibit autophagosome maturation by inhibiting TRAF6 from ubiquitinating a key autophagic protein Beclin-1 (24), we determined the effects of AAT on A20 expression in macrophages. THP-1 cells were infected with *M. intracellulare* ± 1 to 5 mg/mL AAT for 24 h and the whole cell lysates were immunoblotted for A20. Similar to that seen with NFκB binding, *M. intracellulare* strongly increased A20 protein expression that was inhibited by AAT in a dose-dependent fashion (Figure 3E).

**Figure 4:**
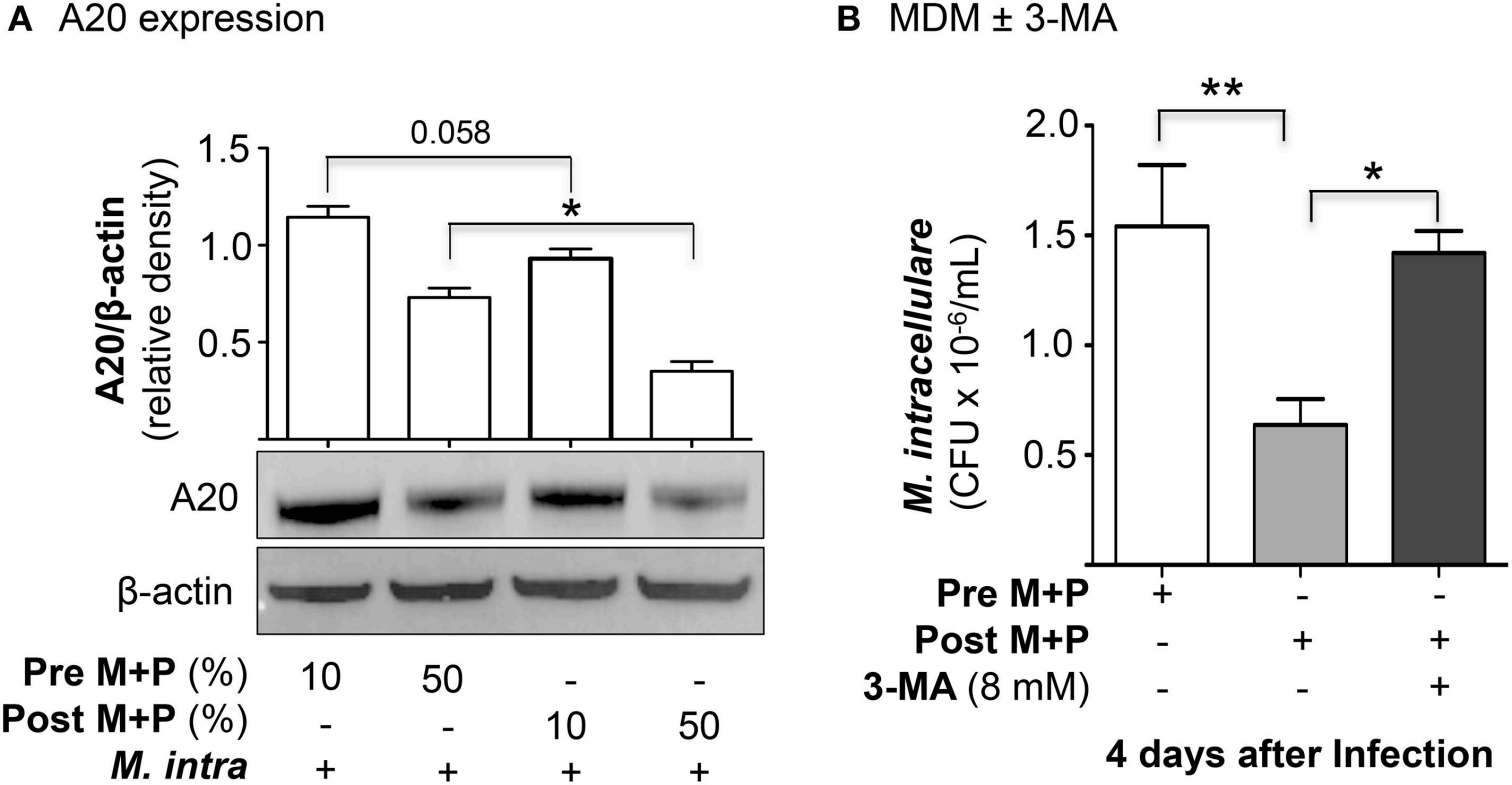
AAT-induced autophagy enhances macrophage control of *M. intracellulare*. **(A)** Pre- and post-AAT infused MDM and contemporaneous 10 or 50% plasma were infected with *M. intracellulare* for 24 h, nuclear-free cell lysate separated by SDS-PAGE, and immunblotted for A20. Data represent the mean ± SEM of cells from three subjects in duplicates. **(B)** Pre- and post-AAT infused MDM and plasma were infected with *M. intracellulare* for 1, 2, and 4 days and in a separate aliquot of post-AAT infused MDM + plasma, 8 mM 3-methyladenine (3-MA) was added simultaneously with the infection. Data represent the mean ± SEM of cells from three subjects in duplicates. ^*^p < 0.05, ^**^p < 0.01; Pre M+P, pre-AAT infused MDM + plasma; Post M+P, post-AAT infused MDM + plasma; *M. intra, M. intracellulare*.

To determine whether there is differential A20 expression between the pre- and post-AAT infused MDM + plasma, we prepared MDM and plasma before and after a session of AAT infusion from three PiZZ subjects and infected the MDM + 10 or 50% contemporaneous plasma with *M. intracellulare* for 24 h. Nuclear-free whole cell lysates were prepared, separated by SDS-PAGE, and immunoblotted for A20. As shown in Figure 4A, there was a modest decrease in A20 expression in the post-AAT infused, *M. intracellulare*-infected MDM incubated in contemporaneous 10% plasma as compared to *M. intracellulare*-infected pre-AAT infused MDM + 10% plasma; the mean densitometry measurements normalized for β-actin showed near significance (*p* = 0.058). There was a significant decrease in A20 expression with the *M. intracellulare*-infected post-AAT infused MDM + 50% plasma compared with the pre-AAT infused *M. intracellulare*-infected MDM + 50% plasma (Figure 4A); one caveat to bear in mind is that the AAT formulation infused into patients also contains proteins other than AAT.

To analyze more definitively if the reduced CFU seen with post-AAT infused MDM and plasma was due to increased autophagy, we performed the *M. intracellulare*-infected MDM experiments with and without an inhibitor of autophagy. From three subjects receiving AAT infusion, we infected pre- and post-AAT infused MDM + plasma with *M. intracellulare* for 1 h, 2 and 4 days; separate aliquots of post-AAT infused MDM + plasma were infected with *M. intracellulare* with or without simultaneous addition of 3-methyladenine (3-MA), an inhibitor of autophagosome formation through inhibition of class III phosphatidylinositol-3-kinases (25). 3-MA has no direct effect on mycobacteria (26). Compared to the pre-AAT infused MDM + plasma, there was reduced CFU of *M. intracellulare* at two and four days after infection with the post-AAT infused MDM + plasma (Figure 4B). But with the addition of 8 mM 3-MA to the *M. intracellulare*-infected post-AAT infused MDM + plasma, there was abrogation of this host-protective effect of infused AAT. These findings support the paradigm that the host-protective effect of AAT is due, in part, to induction of autophagy.

### AAT Infusion Differentially Affects Macrophage Cytokine Expression

**Figure 5:**
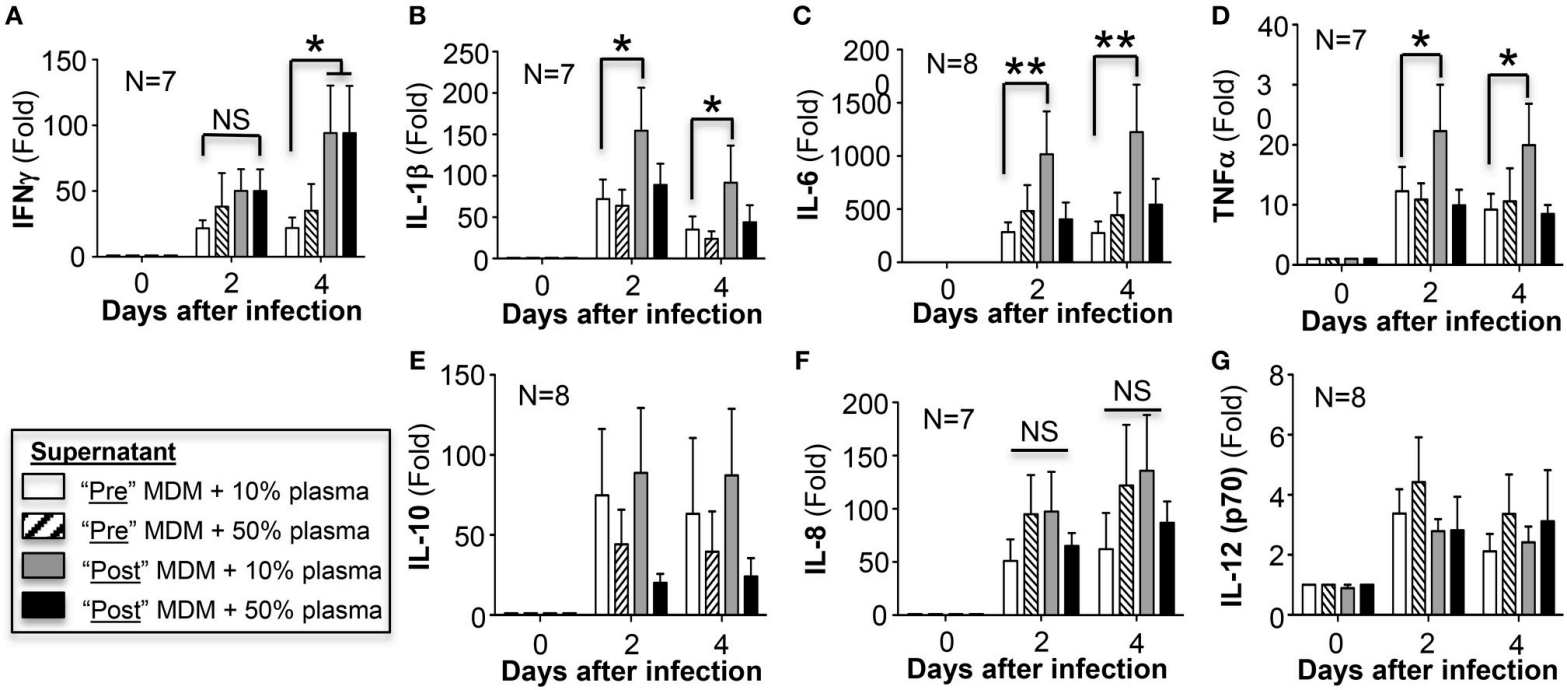
Quantitation of cytokines in the cell culture supernatants. At the indicated conditions and times of infection, the cell culture supernatant of the MDM were assayed for **(A)** IFNγ, **(B)** IL-1β, **(C)** IL-6, **(D)** TNFα, **(E)** IL-10, **(F)** IL-8, and **(G)** IL-12 (p70). Data shown are the mean ± SD of cells from 7 to 8 subjects performed in duplicates. ^*^p < 0.05, ^**^p < 0.01, NS, non-significant.

The concentrations of a panel of secreted pro-inflammatory cytokines as well as IL-10 were measured in the culture supernatant of the same *M. intracellulare*-infected pre- and post-AAT infused MDM/plasma shown in Figure 1B. A modest but significant increase in IFNγ was secreted by MDM at 4 days after infection in the presence of either 10 or 50% plasma obtained after AAT infusion compared to MDM and plasma obtained before infusion (Figure 5A). Post-AAT infused MDM plus 10% plasma showed a significant increase in IL-1β, IL-6, and TNFα compared to their respective MDM and plasma obtained before AAT infusion; however, there was no difference in the levels of these three cytokines between pre- and post-AAT infused MDM when 50% plasma was used (Figures 5B–D). In contrast to the pro-inflammatory cytokines, there was a non-significant trend toward *decreased* IL-10 levels in MDM with 50% plasma obtained after AAT infusion (Figure 5E). There was no difference in the levels of IL-8 or IL-12p70 with any of the conditions (Figures 5F,G).

### AAT Infusion Increased Apoptosis of *M. intracellulare*-Infected MDM

**Figure 6:**
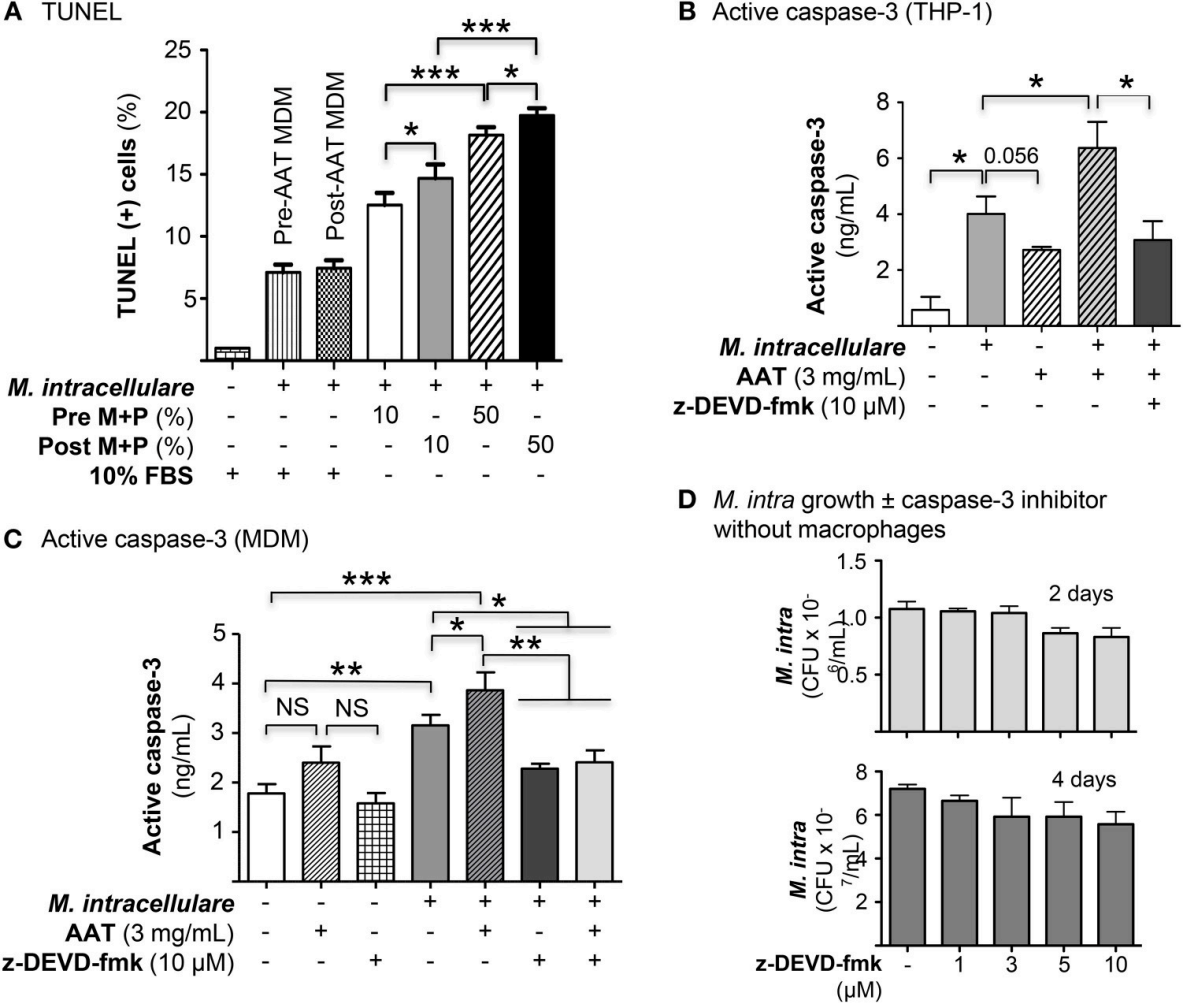
AAT induced apoptosis of macrophages infected with *M. intracellulare*. **(A)** MDM plus 10 or 50% plasma obtained pre- and post-AAT infusion were infected with *M. intracellulare*, incubated for two days with either 10% FBS or 10 or 50% pre- and post-AAT infusion plasma from PiZZ subjects, and TUNEL assay performed. Data shown are the mean ± SEM of cells from eight subjects performed in duplicates. **(B)** Differentiated THP-1 cells were infected with *M. intracellulare* alone or with AAT or with both AAT and z-DEVD-fmk for 24 h, lysed, and activated caspase-3 quantified by ELISA. Data represent the mean ± SEM of three independent experiments performed in duplicates. **(C)** Caspase-3 activity was measured in pre-AAT infused MDM in unstimulated cells or cells infected with *M. intracellulare* alone or with AAT ± z-DEVD-fmk for 24 h. Data represent the mean ± SEM of cells from three subjects in duplicates. **(D)** Liquid cultures of *M. intracellulare* at 5 × 10^5^ mycobacteria per mL were incubated with 0.1% DMSO or 1, 3, 5, and 10 μM z-DEVD-fmk for 2 and 4 days, followed by quantitation of viable *M. intracellulare*. Data represent the mean ± SEM of three independent experiments performed in duplicates. ^*^p < 0.05, ^**^p < 0.01, ^***^p < 0.001, NS, non-significant; Pre M+P, pre-AAT infused MDM + plasma; Post M+P, post-AAT infused MDM + plasma; *M. intra, M. intracellulare*.

The role of apoptosis of infected phagocytes in controlling intracellular NTM is controversial and its role may depend, in part, on the cell type (27–29). Since AAT is known to inhibit apoptosis of epithelial and endothelial cells (30–32), we quantified apoptosis following *M. intracellulare* infection of MDM cultured in autologous plasma obtained before and after AAT infusion. In MDM obtained pre- and post-AAT infusion and cultured in 10% FBS, *M. intracellulare* infection modestly but significantly increased TUNEL positivity (Figure 6A). However, in MDM incubated with autologous plasma and infected with *M. intracellulare*, there was a further increase in TUNEL positive cells, particularly with MDM and plasma obtained after AAT infusion and with greater plasma concentration (Figure 6A). *M. intracellulare* and AAT also increased caspase-3 activity in THP-1 cells, which was inhibited by a caspase-3 inhibitor, z-DEVD-fmk (Figure 6B). Interestingly, compared to THP-1 cells, pre-AAT infused MDM have higher basal level of caspase-3 activity and while addition of exogenous AAT further increased caspase-3 activity, the increase was not statistically significant (Figure 6C). However, infection with *M. intracellulare* significantly increased caspase-3 activity, which was further increased with both *M. intracellulare* and AAT, and reduced to near basal level with addition of z-DEVD-fmk (Figure 6C). Prior to elucidating whether caspase-3 dependent classical apoptosis is host protective, deleterious, or neutral, we ascertained whether z-DEVD-fmk influences the viability and growth of *M. intracellulare* in the absence of macrophages. Separate liquid cultures of 5 × 10^5^/mL of *M. intracellulare* were incubated with 0.1% DMSO vehicle or 1, 3, 5, or 10 μM z-DEVD-fmk for 2 and 4 days. As shown in Figure 6D, there was an insignificant reduction in CFU with the two highest concentrations of z-DEVD-fmk.

**Figure 7:**
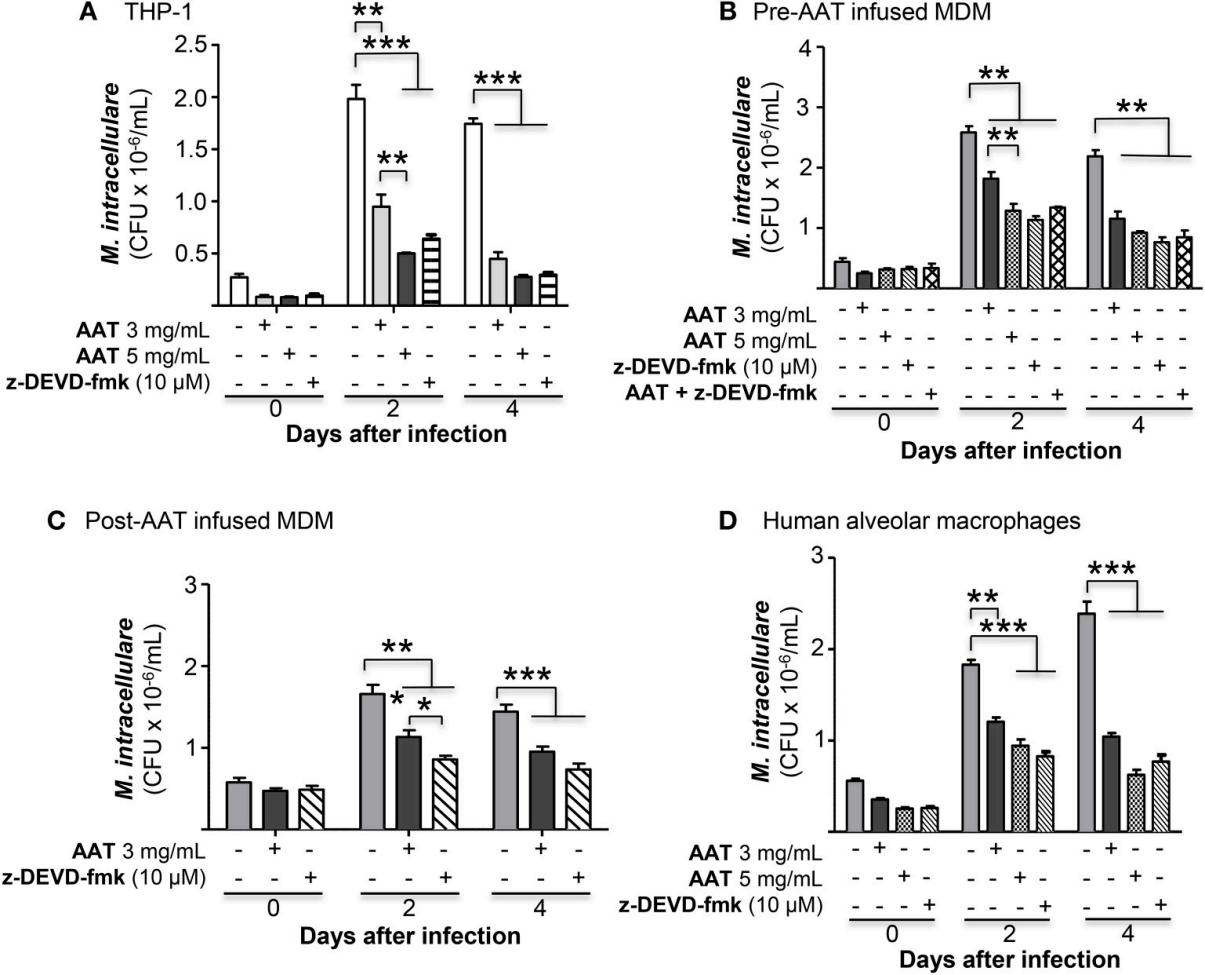
Inhibition of caspase-3 reduced *M. intracellulare* burden in macrophages. **(A)** *M. intracellulare* growth in THP-1 cells ± AAT or caspase 3 inhibitor z-DEVD-fmk (10 μM) at 1 h, 2, and 4 days after infection. Data shown are the mean ± SEM of five independent experiments performed in duplicates. **(B)** MDM derived from pre-AAT infused monocytes from PiZZ patients incubated with 10% FBS were infected with *M. intracellulare* alone or with AAT (3 or 5 mg/mL), 10 μM z-DEVD-fmk, and both 5 mg/mL AAT and 10 μM z-DEVD-fmk. After the indicated times, cell-associated *M. intracellulare* were quantified. Data shown are the mean ± SEM of cells from four subjects performed in duplicates. **(C)** MDM derived from post-AAT infused monocytes and plasma incubated with 3 mg/mL exogenous AAT or 10 μM z-DEVD-fmk and infected with *M. intracellulare* for the indicated times and cell-associated *M. intracellulare* quantified. Data shown are the mean ± SEM of cells from three subjects performed in duplicates. **(D)** Primary human alveolar macrophages from organ donors were cultured as described in Experimental Methods and incubated with AAT or z-DEVD-fmk at the indicated concentrations for 1 h, and 2 and 4 days, followed by quantitation of *M. intracellulare*. Data shown are the mean ± SEM of cells from six cadaveric donors performed in duplicates. ^*^p < 0.05, ^**^p < 0.01, ^***^p < 0.001.

To investigate whether apoptosis of *M. intracellulare*-infected macrophages may help to eliminate intracellular NTM, THP-1 cells were infected with *M. intracellulare* alone or with AAT (3 or 5 mg/mL) or 10 μM z-DEVD-fmk and the bacterial burden quantified at 1 h, 2 and 4 days. As shown in Figure 7A, addition of either AAT or caspase-3 inhibitor significantly reduced the burden of cell-associated *M. intracellulare*. Thus, contrary to our expectations, apoptosis does not enhance but rather impairs macrophage control of *M. intracellulare* infection. To confirm this finding in primary cells, MDM derived from pre-AAT infused monocytes incubated with 10% FBS were also infected with *M. intracellulare* alone or with AAT (3 or 5 mg/mL), 10 μM z-DEVD-fmk, and both 5 mg/mL AAT and 10 μM z-DEVD-fmk. We found that addition of exogenous AAT significantly reduced cell-associated *M. intracellulare* (Figure 7B), consistent with the findings seen with the THP-1 cells and the pre- and post-AAT infused MDM and plasma experiments. Similar to that seen with THP-1 cells, inhibition of caspase-3 activity significantly reduced the burden of *M. intracellulare* (Figure 7B). There was no additive or synergistic effect of AAT and z-DEVD-fmk (Figure 7B). Similarly, addition of exogenous AAT to post-AAT infused MDM reduced cell-associated *M. intracellulare*; caspase-3 inhibition did not reverse the control of *M. intracellulare* infection but in fact further reduced the cell-associated bacterial burden (Figure 7C). To validate that this phenomenon is also seen with lung cells, we infected primary human alveolar macrophages with *M. intracellulare* alone, or with either AAT (3 or 5 mg/mL) or z-DEVD-fmk. Qualitatively similar to that seen with both THP-1 cells and MDM, infection of the alveolar macrophages also resulted in a productive infection but there were significant reductions of cell-associated *M. intracellulare* with addition of either AAT or z-DEVD-fmk (Figure 7D). While z-DEVD-fmk reduced the number of viable *M. intracellulare* slightly in the absence of macrophages (Figure 6D), the effect of z-DEVD-fmk on reducing macrophage-associated *M. intracellulare* was significantly more profound, further supporting the notion that apoptosis impairs macrophages in controlling cell-associated *M. intracellulare*.

## Discussion

We compared the ability of MDM and plasma isolated from 10 PiZZ subjects pre- and post-AAT infusion to control an *ex vivo M. intracellulare* infection. The combination of post-AAT infused MDM and plasma—when total and M-AAT plasma levels are at their peak—was significantly better able to control *M. intracellulare* infection compared to pre-AAT infused MDM and plasma, when plasma AAT levels were at their nadir. Because *M. intracellulare* burden was lower in both MDM and THP-1 cells cultured in the presence of 50% as compared to 10% plasma—even before AAT infusion when AAT levels in the culture medium are well-below normal blood levels—it suggests the possibility that other plasma component(s) in addition to AAT may also enhance MDM control of the infection. For example, the secretory leukocyte protease inhibitor (SLPI) has been shown to inhibit degradation of opsonins and receptors involved in phagocytosis (33). But there was no significant difference in the bacterial load at 1 h after infection with pre- and post-AAT infused MDM and plasma, making it unlikely that AAT preserved opsonization and increased phagocytosis. An unproven mechanism for the host-protective effect of M-AAT against *M. intracellulare* may be that AAT antagonizes NTM components—as has been shown for an AAT variant that antagonizes *Pseudomonas* exotoxin A (34). But our finding of the lack of a direct toxic effect of AAT on *M. intracellulare* would make this hypothesis less tenable.

**Figure 8:**
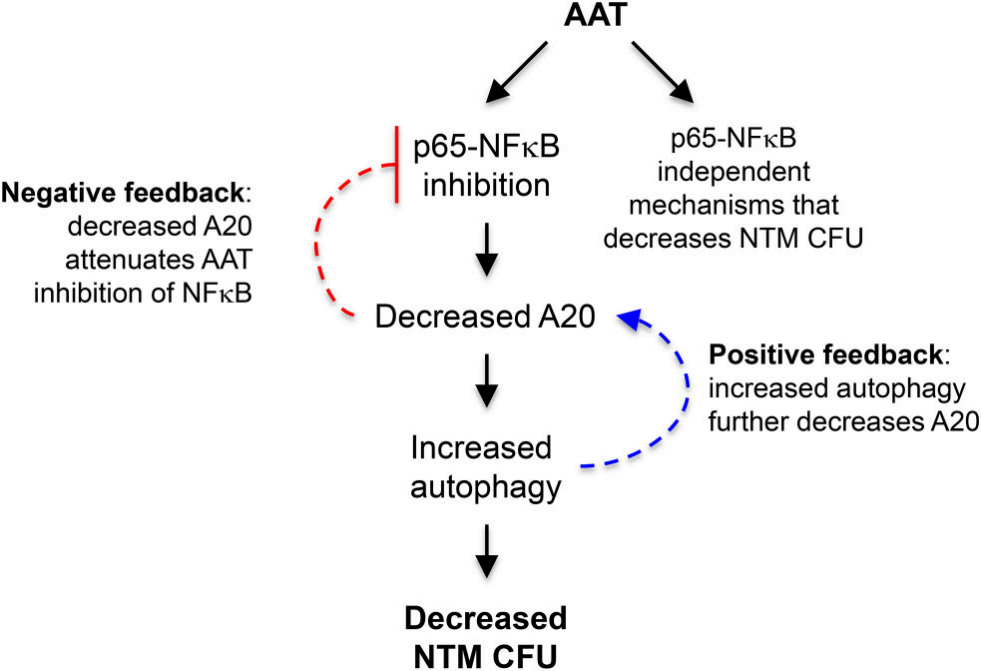
Cartoon of the mechanism by which AAT enhances macrophage control of *M. intracellulare infection*. The paradigm from the results of this study is that AAT inhibits p65-NFκB activation in *M. intrcellulare*-infected macrophages, resulting in inhibition of autophagy and increased susceptibility to *M. intracellulare* infection in macrophages. It is also likely that AAT is inducing other host-protective mechanisms in macrophages that are independent of p65-NFκB activation. Based on the work of others, increased autophagy may further decrease A20, further amplifying autophagy and providing a positive feedback loop (dashed blue arrow); however, decreased A20 expression may attenuate AAT inhibition of NFκB, preventing excessive inhibition of NFκB by AAT and displaying a negative feedback loop (dashed inhibitory red arrow).

Post-AAT infused MDM and plasma infected with *M. intracellulare* have increased phagosome-lysosome fusion and autophagosome formation and maturation—due to AAT inhibition of the expression of A20, a known inhibitor of autophagy—compared to MDM and plasma obtained before AAT infusion (Figure 8). We further confirmed that inhibition of autophagy with 3-MA abrogated the anti-*M. intracellulare* effects of AAT in macrophages. While addition of exogenous AAT to either THP-1 cells or MDM inhibited *M. intracellulare*-induced NFκB activation, there was no such difference between infected pre- and post-AAT infused MDM, making it unlikely that inhibition of p65-NFκB by AAT exclusively mediates the increased autophagy and reduced bacterial burden in the post-AAT infused conditions. We speculate that other p65-NFκB independent mechanisms that may account for post-AAT infused MDM and plasma possessing greater control of *M. intracellulare* infection include: (i) AAT inhibition of NFκB subunits other than p65, (ii) other effector mechanisms against NTM such as phagosome-lysosome fusion that is independent of NFκB activation, (iii) basal differences between THP-1 cells and MDM in the kinetics and subtypes of NFκB that may be activated by mycobacterial infection, a plausible hypothesis since we previously saw qualitative differences in the activation of different NFκB isoforms in THP-1 cells, MDM, and primary human alveolar macrophages infected with *M. tuberculosis* (17), and/or (iv) other as-yet-unidentified plasma components contained in the infused AAT that also antagonizes *M. intracellulare*; i.e., pharmacologic AAT products contain other plasma components. Nevertheless, our findings are consistent with previous work showing that inhibition of NFκB activation increases autophagy (17).

Bergin et al. (35) showed that AAT binds to extracellular IL-8, preventing the latter from engaging its receptor CXCR1 and activating Akt. Since Akt activation inhibits early signaling pathway for autophagy, perhaps AAT binding to IL-8 prevented full activation of Akt thereby inducing autophagy (36). However, because there was no difference in IL-8 measured in the supernatant of MDM plus plasma pre- and post-AAT infusion, this hypothesized mechanism for AAT induction of autophagy is less plausible. Furthermore, since polymers of Z-AAT has been shown to activate NFκB (37, 38) and conversely, normal M-AAT inhibits NFκB activation (12, 20), we posit that the increased autophagy seen with the post-AAT infused MDM and plasma was not only due to the higher M-AAT concentration but also to a relatively lower Z-AAT concentrations. Aldonyte et al. (23) found that sub-physiologic concentrations of native or polymerized AAT (~0.1 mg/mL) induced NFκB activation whereas more physiologic concentrations (0.5 to 1 mg/mL) of either AAT conformations suppressed NFκB activation in human monocytes, paralleled by induction and repression of pro-inflammatory cytokines, respectively. The related protease inhibitor SLPI has been shown to inhibit NFκB activation by preventing degradation of IκBα (33).

Thus, our paradigm is that AAT inhibition of NFκB activation and A20 expression increased autophagy, reducing intracellular NTM number (Figure 8). AAT inhibition of A20 expression is most likely secondary to inhibition of NFκB activation since NFκB is a potent transactivator of A20 gene expression (39, 40). As previously mentioned, AAT is likely inducing other host-protective mechanisms in macrophages that are independent of p65-NFκB activation (Figure 8). In addition, others have shown that autophagy decreases A20 by sequestering it in autophagosomes, resulting in the inhibition of NFκB activation as A20 is also an inhibitor of NFκB (41). While this discovery and our own findings are not necessarily contradictory, they suggest the presence of a possible positive and negative feedback loop; *i.e*., the increased autophagy we saw with AAT would further decrease A20 leading to more autophagy (positive feedback loop) (Figure 8, dashed blue arrows) but the decreased A20 would attenuate AAT inhibition of NFκB (negative feedback loop) (Figure 8, dashed inhibitory red arrow). This complex level of control of NFκB activation may be necessary because NFκB has both host-protective and host-deleterious effects and which function dominates likely depends, in part, on its level and timing of activation as well as possibly the specific NFκB isoform(s) being activated.

IFNγ was the one cytokine in which there was a consistent increase in MDM plus 10 or 50% plasma obtained post-AAT infusion compared with their respective pre-AAT infused MDM and plasma. Post-AAT infused, *M. intracellulare*-infected MDM and 10% plasma produced significantly more IL-1β, IL-6, and TNFα compared to similarly infected MDM plus pre-AAT infused plasma. While it appears puzzling that M-AAT inhibits NFκB activation and yet there was increased level of some pro-inflammatory cytokines with post-AAT infused MDM + 10% plasma, this apparent paradox is also exemplified by nicotine since nicotine activates NFκB as well as inhibits pro-inflammatory cytokine production through induction of various microRNAs that inhibit cytokine protein expression (18). Perhaps AAT may be inducing some cytokines through such indirect mechanism. It is noteworthy that IL-10—an immunosuppressive cytokine known to predispose to mycobacterial infections (42–44)—achieved the lowest level in the post-AAT infused MDM and 50% plasma. While it is reasonable to posit that this reduction of IL-10 may be responsible for increased pro-inflammatory cytokines, the greatest reduction of IL-10, was seen with the *M. intracellulare*-infected, post-AAT infused MDM and 50% plasma, whereas the greatest increase in pro-inflammatory cytokine production was with the post-AAT infused MDM and 10% plasma; i.e., the higher IL-1β, TNFα, and IL-6 seen with *M. intracellulare*-infected post-AAT infused MDM + 10% plasma is difficult to attribute to reduced IL-10. An alternative but not mutually exclusive possibility for the lower IL-1β, IL-6, and TNFα production with the post-AAT infused 50% plasma compared to 10% plasma is that the higher plasma concentration of M-AAT with the former resulted in greater competition with the Z-AAT since Z-AAT is known to induce NFκB activation and pro-inflammatory cytokines (12, 20, 45–48). Indeed, dilution of whole blood with cell culture medium but not with normal plasma increased the spontaneous production of several but not all pro-inflammatory cytokines (49). In support of our findings of higher IL-1β, TNFα, and IL-6 with the lower concentration of post-AAT infused plasma, others have found that sub-physiologic AAT concentrations (~0.1 mg/mL) significantly induced pro-inflammatory cytokines such as TNFα, IL-6, and IL-8 to a greater degree than higher physiologic AAT concentrations in human monocytes (23).

In contrast to reports that AAT inhibits apoptosis of endothelial and epithelial cells (30, 32), we found an increase in apoptosis of post-AAT infused MDM/plasma that were infected *ex vivo* with *M. intracellulare* as compared to pre-AAT infused MDM/plasma infected with the NTM. Since A20 is not only anti-autophagic but also anti-apoptotic (24), AAT inhibition of A20 may be, in part, responsible for the increased apoptosis seen with post-AAT infused plasma of *M. intracellulare*-infected macrophages. Furthermore, based on the caspase-3 inhibitor studies in THP-1 cells, MDM, and primary human alveolar macrophages, it would indicate that apoptosis of *M. intracellulare*-infected macrophages is a disadvantage to the host; i.e., post-AAT infused MDM and plasma are better able to control *M. intracellulare* infection despite inducing apoptosis of the infected macrophages. While apoptosis of *M. tuberculosis*-infected macrophages is generally considered to be a host-protective effector mechanism (50–52), apoptosis has also been shown to facilitate cell-to-cell spread of the organisms (53, 54). Similarly, in NTM-infected macrophages, both salubrious and deleterious roles of apoptosis have been described (27–29, 55). One caveat of these apoptotic studies is that caspase-3 may have activities that are independent of classical apoptosis. Chakravortty et al. (56) showed that caspase-3 inhibition with two different caspase-3 inhibitors (z-VAD-fmk and z-DEVD-fmk) abrogated lipopolysaccharide-induced NFκB activation in a mouse macrophage cell line, with the implication that caspase-3 activates NFκB. Thus, it is possible that the reduced *M. intracellulare* CFU seen with the z-DEVD-fmk may be due, in part, to this phenomenon.

What is the potential clinical significance of these findings? Certainly it would suggest that in those with frank AAT deficiency, AAT replacement may not only help delay the progression of their emphysema but may also be a potential adjunctive agent to the antibiotics used to treat a concomitant NTM lung infection. However, because AAT is an acute phase reactant, it remains to be determine whether those without deficiency but perhaps a “suboptimal” level—which remains to be defined—may also benefit from AAT as a pharmacologic agent as has been suggested for cystic fibrosis patients (57). Moreover, in illnesses where there is excessive oxidative stress, AAT levels may be adequate and yet the protein dysfunctional due to oxidation of methionine 351 or 358 (58). Thus, it remains to be investigated whether addition of exogenous AAT to patients without deficiency would be beneficial.

In summary, AAT may not only protect against NTM-LD through preventing the development of emphysema and bronchiectasis, but also through enhancing macrophage immunity against NTM. Such host-protective AAT-induced mechanisms include induction of phagosome-lysosome fusion, autophagy, and host-protective cytokines. Some AAT-induced intracellular events that may mediate these macrophage effector functions include AAT inhibition of NFκB activation and A20 expression. Further *in vivo* animal studies and use of primary macrophages and other cell types from PiZZ subjects in which only the abnormal *SERPIN1* gene is replaced with the normal *SERPIN1* gene are needed to further validate these findings.

## Ethics Statement

Human Subject study was approved by the Colorado Multiple Institutional Review Board (Protocol Number 14-0755) and all recruited patients signed informed consent.

## Author Contributions

AB, XB, and EC: conception and design. XB, AB, JH, CE, GH, RH, BK, RS, and EC: analysis and interpretation. XB and EC: wrote first draft. XB, AB, and EC: wrote sections of the manuscript. XB, AB, JH, CE, AM, ZF, GH, RH, BK, RS, and EC: manuscript review, read, and approved.

### Conflict of Interest Statement

The authors declare that the research was conducted in the absence of any commercial or financial relationships that could be construed as a potential conflict of interest.
